# Closing the Loop: Enhancing Local Monitoring of Child Poverty to Leave No Child Behind

**DOI:** 10.3390/children11010067

**Published:** 2024-01-05

**Authors:** Pablo de la Rasilla, Iraklis Stamos, Paola Proietti, Alice Siragusa

**Affiliations:** 1 Independent Researcher, 1000 Brussels, Belgium; pablodelarasilla@gmail.com; 2 Territorial Development Unit, Joint Research Centre, European Commission, 41092 Seville, Spain; alice.siragusa@ec.europa.eu; 3 Territorial Development Unit, Joint Research Centre, European Commission, 21027 Ispra, Italy; paola.proietti@ec.europa.eu

**Keywords:** child poverty, local monitoring, sustainable development goals, vulnerable children, equitable and inclusive societies, Agenda 2030

## Abstract

Research on the Leave No One Behind principle of the Sustainable Development Goals (SDGs) within the context of the Agenda 2030 is currently prevalent; however, research on monitoring child poverty at the sub-national (local) level is still limited. This paper addresses this gap by examining indicators developed for monitoring the phenomenon at different territorial levels (global, European, and national) and assessing their territorial transposition locally, using the city of Cadiz, Spain, as a case study. Interviews with local stakeholders reveal that despite the availability and access to related indicators and data, relevant actors must enhance their efforts to utilize such indicators effectively. Based on desktop research and qualitative analysis, the paper delivers recommendations for improving local monitoring of child poverty in Europe and inducing policy changes. This knowledge can inform targeted interventions, policy formulation, and resource allocation to tackle child poverty and promote equitable and inclusive societies.

## 1. Introduction

Child poverty is an enduring social issue closely linked to wealth distribution among different regions and socioeconomic groups. It is also rooted in the inherent vulnerability of children who have not yet reached the age of 18. Children living in poverty mostly depend on their families and, unlike other age groups, are often unable to overcome their financial hardship or its consequences independently. Poverty can devastate children by depriving them of basic needs such as food, shelter, and healthcare [1]. It can prevent them from accessing the resources necessary for personal development, including education and safe environments. Such adverse circumstances have a lasting impact on children and can lead to poor health outcomes, limited academic success, and fewer economic opportunities in adulthood [2]. The consequences of childhood poverty are well documented in the literature [3]: children exposed to poverty can experience long-term harm, including wasting, stunting, and physical and emotional abuse or neglect. As such, child poverty is a timeless, poignant issue that has significant ramifications for individuals and society as a whole. The impact and consequences of this global issue cut across geographical boundaries, affecting regions worldwide, albeit with varying degrees of intensity. Child poverty is not only present in emerging economies but also in advanced ones [4]; across the OECD countries, almost one out of six children (on average) live in poverty, i.e., children that are excluded from the minimum acceptable way of life because of limited material, cultural, and social resources [5].

Precisely because of the vulnerability and the long-term consequences of child poverty, the UN adopted Agenda 2030 and its Sustainable Development Goals (SDGs) have recognized child poverty as a significant issue and have taken measures to address it through Goal 1, which aims to eradicate poverty, including child poverty, in all its forms and dimensions. Moreover, Goal 4 focuses on providing inclusive and equitable education to children in poverty-stricken areas to ensure their well-being and promote economic and social mobility. The SDGs framework includes a series of indicators for directly and indirectly monitoring child poverty. Such indicators concern for example the percentage of children living in households with income below the poverty line, the proportion of children enrolled in primary and secondary education, or the percentage of children with access to essential vaccines and healthcare services. These indicators are crucial for tracking progress towards eradicating child poverty and achieving the SDGs’ vision of a world free from poverty and inequality. Similarly, at the European level, the European Commission has lately intensified its commitment to addressing child poverty by establishing the European Child Guarantee in 2021 and monitoring child poverty through indicators such as severe material and social deprivation.

Despite the growing political and societal acknowledgement of the impact of child poverty, and an overall amplified effort to accurately quantify and document the phenomenon’s actual scale, the reality still uncovers limitations in child poverty monitoring and data collection [6]. The latter gains in importance when accounting for the territorial level of analysis of child poverty, where the sub-national level is largely disregarded [7]. Although consolidated statistical indicators (such as the ones of the UN suggested SDG framework) provide a context for measuring child poverty, they are conceived and designed to account for the national level. However, deploying an approach of measuring a largely local phenomenon (growingly present in agglomerations with high population density, for instance, cities and regions) entails the risk of masking inequalities and not accurately reflecting reality for example by disregarding the disaggregating aspects of the phenomenon (e.g., regarding ethnicity, migratory status or gender); and thus, taking less-informed decisions and possibly designing less-well matching policies and actions to address the phenomenon.

To address the need for knowledge (in policy and pertinent literature) on how child poverty is monitored at the local level and explore the potential of what such information would have against current monitoring practices (in terms of indicators used for measuring the phenomenon), this paper aims to compile and consolidate existing knowledge in the field, by identifying practices reported in the literature, and research on how local specificities of child poverty can be captured and reflected in relevant indicators. It does so, among others, by following a case-study (focusing on a mid-sized European city) and a participatory approach, allowing to integrate local actors and stakeholders knowledge and perception of the phenomenon in the assessment.

The remainder of the paper is structured as follows: Section 2 reviews the pertinent literature related to child poverty monitoring, with a greater emphasis on the local level aiming to understand the overall framework within which the phenomenon is monitored in cities, municipalities and other urban agglomerations. Section 3 presents the methodological framework adopted herein to address the research question, introduces the case study and the participatory approach adopted including on-field interviews and focus groups with stakeholders dealing with child poverty monitoring at the local level. Section 4 discusses and analyses the findings and results of the paper, while Section 5 presents the conclusions of the conducted research and the formulation of recommendations for future research and policy work in the field.

## 2. Monitoring Child Poverty: Review of Pertinent Literature

Monitoring child poverty at the local level is increasingly important for several reasons. Firstly, child poverty is a complex and multifaceted issue that can vary significantly across different regions and communities. Local-level monitoring enables a deeper understanding of children and families’ unique challenges within specific areas, allowing for targeted interventions and tailored policy responses. Moreover, local-level data provides policymakers and stakeholders with a granular view of poverty dynamics, highlighting disparities and inequalities that may need to be apparent at the national or regional levels. By monitoring child poverty locally, decision-makers can make more informed and evidence-based decisions to allocate resources and implement effective social programs. This is particularly relevant, for example, regarding the need to increase child poverty measurement and monitoring, not only at national and regional levels but also at the local level, where more targeted community programs, housing assistance, education and skills-building programs can be developed. According to the literature, there has not been any notable emergence of new gaps regarding monitoring child poverty at the local level, potentially telling of the marginal progress made in the area since the early 00s. [8] point out that challenges were brought to the forefront at the beginning of the 21st century. Similarly, advancement can be appreciated by using a participatory approach: this would entitle considerations and opinions from children living in poverty that could provide valuable insights for decision-making.

In the literature, various methods are reported to monitor child poverty at all territorial levels, including the local one, each with its own strengths and limitations. One commonly used approach is survey-based data collection, where households within a specific locality are surveyed to gather information on income, household composition, access to essential services, and other relevant indicators [9]. Another method is administrative data analysis, which involves utilizing existing administrative records from social welfare programs, educational institutions, and health services to derive indicators of child poverty [10,11]. By linking and analyzing these datasets, a more comprehensive picture of child poverty at the local level can be obtained even if this is not allowed in all countries. Additionally, qualitative methods such as interviews [12,13], focus groups [14,15] and participatory approaches [16,17] have been used to provide valuable insights into the experiences and perspectives of children and families living in poverty, capturing nuanced dimensions that quantitative data may overlook.

Derived of or feeding into the methods approach when monitoring child poverty at the local level, a range of indicators is used in the literature to assess the well-being and living conditions of children. These indicators include for example measures of income and material deprivation, such as household income [18]; access to essential services [19]; housing quality [20]; and food security [21]. Educational indicators, such as school attendance [22]; dropout rates and educational attainment [23], can shed light on the impact of poverty on children’s access to education. Health-related indicators, such as infant mortality rates [24], malnutrition prevalence and access to healthcare services [25], provide insights into the physical well-being of children within a specific locality. Furthermore, social inclusion indicators, such as social networks [20], community engagement [26], and participation in extracurricular activities, can reflect the social dimensions of child poverty, highlighting the importance of social integration and belonging. For several of those indicators, data are derived from national living conditions surveys, which are then territorially disaggregated, and used to create a snapshot of child poverty at the local level.

Various publications have also shown how important it is to use multidimensional approaches. Initiated by Sen in the 80s, child poverty is not to be studied primarily by the net income indicators for identifying children in poor households, or single other dimensions, but instead via the actual satisfaction of all their primary needs [27]. In that direction, at the institutional level, UNICEF proposes a 2-step multidimensional method to measure child poverty: firstly, by identifying deprivations in several dimensions with reliable indicators for each of them (for example, concerning clothing, education, health, housing, information, nutrition, play, sanitation, and water). Secondly, by measuring the number of people (counting or aggregating) exposed at the same time to several of these dimensions of poverty [28]. Exactly because of its multidimensionality, the UNICEF approach can account for the monitoring (and measurement) of several sub-groups within the child poverty cluster, for example children living in the street, child-headed households, children in institutional care, children staying in correctional facilities, and children in trafficking [29]. Table 1 summarizes indicators reported in the literature that are or can be used for monitoring child poverty, focusing on different dimensions and disaggregations (for all territorial levels).

When measuring child poverty locally, it is vital to consider a range of indicators specific to the context, the population being studied, and the dimensions of poverty. The measures enumerated above provide an overview of some core indicators that can be used to measure child poverty. However, it is worth highlighting some crosscutting issues that may affect intergenerational poverty among children, such as horizontal inequality, social exclusion and discrimination [30]. Horizontal inequality arises when people with similar characteristics differ in access to essential services or their social and economic well-being levels. This can arise, for instance, due to differences in race/ethnicity, religion, geographic location, gender or some other characteristics/status (e.g., disability). It may increase child poverty, specifically when children belonging to specific groups of people are systematically disadvantaged and excluded from essential societal resources such as education, health, and nutritional resources [31]. Social exclusion of vulnerable groups such as women and girls, people living with disabilities, migrants and refugees, and members of ethnic and religious minorities may foster an intergenerational cycle of poverty among children and perpetuate inequalities. This can mainly occur when exclusion and marginalization are meted out in various dimensions of poverty, such as access to education, health and safe housing. These factors may compound the risks of children experiencing poverty, as their basic needs are not satisfied, and their life opportunities and capabilities are curtailed [32]. Discrimination based on gender, race, or other social identity characteristics is also an important consideration when measuring child poverty at the local level. It may compound the risks posed by stereotyping and marginalization. Gender, social and cultural norms also play a role, often, framing children’s (and women) roles primarily within the private sphere, where the provision of unpaid care work and household chores limits their participation in the public sphere. These dynamics can affect children in multiple ways, including less access to health care, education, adequate nutrition, and recreation or play opportunities. In all, effectively integrating horizontal inequalities and cultural norms in the measurement of child poverty is essential to mitigating the global issue of child poverty and helping ensure that all children can live dignified and healthy lives where they can aspire to reach their full potential.

It is essential here to note that the indicators presented and discussed herein, as well as the dimensions of poverty out of which indicators are extracted, are not exhaustive. Another crucial element to consider is that although not with a distinct pattern, the literature suggests that different indicators/dimension might be pertinent in some local contexts and less in others, in or without combination of others. Regardless of which indicators will be chosen to monitor the phenomenon, and synthesizing evidence from the pertinent literature, the key criteria that should be considered and implemented to strengthen the accuracy, comprehensiveness, and timeliness of indicator data collection are common and summarized in Table 2.

These criteria are essential for ensuring that accurate data about child poverty is collected, analyzed, and interpreted to design data-driven policies that are more effective, equitable, and sustainable. However, it is worth noting that some challenges may arise when trying to meet all these requirements in practice. For instance, data collection could be hampered by financing limitations, as data-intensive surveys and other data collection instruments may be resource-intensive and costly to execute. In addition, data collection requires competent and knowledgeable personnel, which may pose a challenge when governments need help attracting and retaining skilled data experts [33]. Moreover, some significant concerns arise when using data around vulnerable or marginalized populations, such as children or specific communities, where data collection and monitoring are typically more challenging to complete than other data sources. These groups may face additional challenges or constraints that reduce their ability to participate in data collection initiatives or access essential resources to improve their life outcomes, making it difficult to collect and analyze representative data that adequately captures child poverty status at the local level but also privacy concerns. To conclude, ensuring data quality and comparability can be challenging and demanding, requiring significant resources and expertise that may not always be available in certain contexts [34]. Hence, the criteria highlighted in the table may only be met in some settings due to insufficient qualified personnel, inadequate resources, poor infrastructure, and financing. Despite these challenges, incorporating these criteria into data collection initiatives is essential for developing effective strategies and policies to mitigate and ultimately eradicate child poverty.

## 3. Materials and Methods

The overall research question of the paper was centered on how child poverty can be monitored at the local level. To answer this question, the authors developed an extensive research methodology that involved multiple stages. The methodology prioritized stakeholder engagement and constructed a participatory framework that captured diverse perspectives while addressing academic rigor. The first step of this process involved a comprehensive desktop review of existing literature on the indicators and methods used for child poverty monitoring (Section 2). Following the desktop review, the authors adopted a case-study approach, focusing on collecting extensive insights from the city of Cadiz, Spain, to offer real-world experiences of child poverty monitoring at the local level. Furthermore, an additional step involved stakeholder mapping, identifying those directly and indirectly involved in the issue to ensure their perspectives were captured and rightly positioned in the framework. Finally, semi-structured interviews were conducted with key stakeholders designed in direct consultation with prior research, extending the literature’s discussion beyond what has already been published. The result was a rich data and information set that enabled the authors to draw meaningful conclusions while highlighting communities’ complexities and challenges when monitoring child poverty in Cadiz and formulating recommendations that could be potentially useful also in other local contexts. This methodology offered a comprehensive perspective of local-level poverty monitoring, ultimately contributing to policy development. Following sections discuss the individual steps of the methodological framework in detail.

### 3.1. A Case-Study Approach

In order to answer the research question on how child poverty can be measured (and thus monitored) at the local level, a case-study approach was followed, similar to that of several pertinent publications [35,36,37,38]. This approach allows for a comprehensive exploration of a specific city’s child poverty situation and at the same time provides an opportunity to examine the unique context, complexities, and dynamics that contribute to child poverty in that particular location. It also enables a deeper understanding of the local factors that may not be evident when conducting broader or cross-sectional studies. By focusing on a specific city, the case-study approach adopted herein, enabled the authors to consider the social, economic, cultural, and political factors that influence child poverty within that particular community. It also allowed for a nuanced analysis of the city’s characteristics, such as local policies, application of institutional frameworks, and available resources, which can shed light on the root causes and potential solutions for child poverty, but predominantly, on how child poverty is monitored and potentially measured. At the same time, following a case-study approach granted the authors access to qualitative data through methods such as interviews, observations, and document analysis that were deployed in this work. In addition, by focusing on a single city, the case study can aim at providing a holistic view of child poverty, taking into account various dimensions beyond income, such as education, healthcare, housing, and social services. In this sense, the approach enabled the authors to explore the interconnections between different factors and how they collectively contribute to child poverty within the specific city context. As a case study can offer valuable insights for policymakers, practitioners, and community stakeholders at the local level, the detailed analysis of child poverty in a specific city can also inform the development and implementation of targeted interventions, policies, and programs that address the unique challenges faced by children and families in that locality, and the findings from a case study can be more actionable and relevant for local decision-making compared to broader studies. Nevertheless, the authors acknowledge that the case-study approach has limitations, in particular regarding potential difficulties in generalizing findings to other contexts and the need to carefully select and define the case to ensure representativeness for the phenomenon of child poverty. Such limitations are further discussed in Section 3.4.

In light of the above, Cadiz, a small-sized Spanish city was selected as a case-study due to several factors. Firstly, Spain is currently grappling with high levels of poverty and social exclusion rates for children living in households, being among the lowest three performing countries according to the AROPE rate in 2019 [39], indicating the need for comprehensive studies and interventions in this area. Additionally, Cadiz is located in Andalusia, the region in Spain with the highest number of people at risk of poverty or experiencing severe material and social deprivation and low work intensity [40]. In addition, data from the Integrated Social Services System (SISS) shows that Cadiz had the highest number of children under the care of the child protection system as of 2021 [41]. In the same year, Cadiz was the Andalusian province with the highest admissions of unaccompanied minors, according to data from the Information System for Minors (SIME) [42]. Recent reports also identify Cadiz as having moderate to low compliance with the SDGs directly linked to child poverty, requiring more attention to the various challenges the city faces [43].

Looking at the within the city level, Cadiz has several disadvantaged neighborhoods, including Barriada de la Paz, Guillen Moreno, Barriada de Loreto, and Barriada Cerro del Moro, which have been classified as disadvantaged based on measures of unemployment, illiteracy, school dropout rates, migrant population, and housing disrepair. These indicators have a direct link to child poverty rates. Finally, the city is home to specific groups of children, often in situations of poverty, such as those from Roma communities, which are prevalent in the neighborhood of Barriada de la Paz. In summary, the city of Cadiz was selected as there were:

- Wealth of reported experiences and practices in the literature on how the local administration monitors and acts on child poverty, allowing for elaboration and analyses, among others, on: indicators used to monitor child poverty; reasons for incomplete data sources; significance of data availability, curation and accessibility; impact of policy actions to address the phenomenon at city level; potential of transferability of findings to other European urban areas [44,45].
- Confirmed availability, interest and willingness of pre-identified stakeholders in sharing insights and experiences on the topic during the scanning of several case-study options.
- Accessibility of the authors to the city. This in practice meant the ability to perform research with stakeholders on the local language; knowledge of the political and administrative landscape, including in the area of social policies and social work in the field; acquaintance of authors with the local cultural specificities and possible nuances, e.g., in regard to the historical and cultural heritage of the city that can influence the city’s social fabric and identity, potentially shaping attitudes and norms related to child poverty [46], or its socioeconomic disparities with certain neighborhoods or areas facing higher levels of poverty.

### 3.2. Stakeholder Mapping

Stakeholder mapping is a widely used process referring to identifying and analyzing the stakeholders who are relevant to a particular research topic or project [47]. It typically includes identifying individuals, groups, organizations, or institutions with an interest or stake in the issue. Within this paper, a stakeholder mapping exercise was conducted to:

- Identify key stakeholders: ensure that the authors engage with the right people during the interview process; avoid missing essential perspectives or insights.
- Determine influence and power dynamics among different stakeholders: Identify potential, influential stakeholders who can shape the research findings or have a significant impact on the issue studied.
- Tailor the interview approach, questions, and communication strategies to engage with each stakeholder effectively: this allows for more meaningful and targeted discussions during the interviews.

For the stakeholder mapping, significant inputs were drawn from a related scientific activity focusing on child poverty in Malaga, Madrid and Bilbao (Spain) [48]. Moreover, for this paper, recommendations for completing the stakeholder mapping exercise were provided by the Coordinator of Social Inclusion of the Spanish European Anti-Poverty Network (EAPN), which were considered. The stakeholder mapping exercise for child poverty was conducted between January and March 2023 and concluded with the following categories of child poverty-related stakeholders for the city of Cadiz:

- Local administration and government.
- Civil servants involved in providing social policies at the local level.
- Social workers in contact with the target group, e.g., in the local children’s hospital.
- Principals of schools in neighborhoods where the child poverty phenomenon is intense within Cadiz.
- Prosecutors of minors intervening in cases of unaccompanied minors and minors whose families lost their custody.
- Representatives of the Andalusian Ombudsman.
- Representatives of academia.
- Civil society organizations, e.g., NGOs, Foundations and Youth Houses.
- International organizations, e.g., the Spanish Red Cross and UNICEF Spain, are involved in projects where monitoring of child poverty is relevant.

The complete list of stakeholders is included in Appendix A.

### 3.3. Semi-Structured Interviews

After identifying the relevant stakeholders through the stakeholder mapping exercise, the authors designed semi-structured interviews to gather insights into the experiences, challenges, and perspectives of individuals directly related with child poverty in Cadiz. The questions asked during these interviews were carefully designed to address gaps identified in the literature through the desktop review, the extended review of indicators and the case study approach and to ensure that a diverse range of perspectives was captured. The interviews were conducted in a language accessible to the participants, either in Spanish or English, both of which the authors spoke fluently. Additionally, the author ensured the anonymity of the participants to protect their confidentiality while conducting the research. This was especially important as subjects were sharing experiences of intimate nature. Throughout each interview, the author remained attentive and engaged with participants, allowing any follow-up questions to be asked and further detailed the participants’ responses. With these interviews, the author could capture the voices of those most involved with child poverty in Cadiz, drawing out insights not evident from desk research of available literature. Ultimately, the data collected from these interviews allowed for the formulation of nuanced and comprehensive responses to the research question of how to measure and monitor child poverty locally.

In total, 31 stakeholders were contacted via email and/or phone call, with 19 agreeing to in-person interviews and 12 agreeing to remote interviews conducted by phone or video call. The interviews for the in-person stakeholders were conducted in the city of Cadiz between the 6 and 8 March 2023, while those run remotely took place between 31 January and 15 April 2023. Six stakeholders could not be interviewed for various reasons, including limited availability during the designated period or missing response to communication attempts. The duration of the interviews was, on average, 1 h. Depending on the prior agreement of the interviewees, the interviews were either recorded and transcribed verbatim (*n* = 17) or notes were taken during the process (*n* = 14). Interview questions were designed to explore the following aspects:

- Interviewees’ knowledge in the area of child poverty;
- Tools deployed to monitor child poverty at the city level;
- How the monitoring and measuring the phenomenon subsequently informs and feeds policy decisions and (if so) how it is used to formulate and/or evaluate local policies targeting child poverty.
- Lessons learnt from current practice, best practices and areas for improvement in monitoring child poverty in the city.

In addition, to the more general question formulated at the beginning of each interview (mentioned before), the following questions were also asked to the stakeholders, aiming at getting more precise information specifically on aspects related with the monitoring:

1. In your experience and capacity, what indicators (if any) are used to monitor child poverty in Cadiz?
  - What data sources are used to measure such indicators?
  - How do you assess the reliability of these data sources?
  - Are indicators used comparable to other cities or regions in Spain or in the EU?
2. At what frequency is child poverty monitored in the city?
  - How often are relevant databases and sources updated?
3. Are specific tools or methods used to monitor child poverty in a more disaggregated manner (e.g., Roma or other ethnicities in child poverty, migrants, gender-based),
  - If yes, please name them.

### 3.4. Limitations

One of the limitations of the approach used in the research paper is the potential difficulty of generalizing the findings to other contexts. The research study focused on Cadiz, Spain, and may not apply to other cities in different regions or countries. Additionally, the stakeholder mapping process may have missed some relevant participants who would have contributed valuable insights into the research question. Consequently, the findings may be limited to the perspectives of the stakeholders involved in the study. Another limitation is the potential for biases in interpreting the collected data. Although efforts were made to minimize biases, such as designing interview questions following a structured methodology, there are still potential biases inherent in qualitative research. The researcher’s perspective may influence how the data is collected, analyzed, and interpreted, potentially skewing the results. Finally, the case-study approach used in the research paper may have limited the generalizability of the findings. Although the approach provided in-depth insights into the complexities of child poverty in Cadiz, Spain, the findings may only apply to some contexts or cities with different socio-economic, cultural and political contexts in certain areas where others influence child poverty.

## 4. Results

The issue of child poverty is multifaceted and can be approached through various indicators such as education, household income, and food security. This section aims to provide a comprehensive overview of the categories and dimensions of children in need, as identified by professionals interviewed in Cadiz. Additionally, it seeks to highlight the indicators used for local-level monitoring. The results presented here illustrate the monitoring of the most vulnerable categories of children in Cadiz, aligning with the SDGs’ Leave No One Behind (LNOB) principle. To enhance readability, the Results section is divided into two parts: The first part focuses on the findings regarding the different categories of vulnerable children identified in Cadiz (aligned with the European Child Guarantee framework). The second part presents the dimensions identified in Cadiz that are essential for monitoring child poverty.

### 4.1. Categories of Vulnerable Children

#### 4.1.1. Children Experiencing Homelessness

Interviewees from the Spanish Red Cross in Cadiz emphasized the significance of conducting local youth surveys to identify vulnerable situations, such as homelessness, and assess children’s living conditions in these circumstances, as for example in [49]. These surveys can employ various indicators to evaluate the housing situation of respondents, including living in homeless shelters, occupied houses (squats) or on the streets. Additionally, data collected from these surveys is disaggregated by gender, origin, age, administrative situation, educational level, and territorial location at the provincial level. Notably, the Red Cross report utilizes the European indicator AROPE and the Spanish Survey of Living Conditions (Encuesta de Condiciones de Vida, ECV) to extract essential information on minors.

#### 4.1.2. Unaccompanied Minors

When addressing unaccompanied minors (UAMs), interviewees stressed the importance of tracking the number of UAMs upon their arrival in Spanish territory and monitoring their development to identify potential indicators of child poverty. The Ministry of the Interior, in collaboration with the Police, Civil Guard, and Public Prosecutor’s Office, maintains a database of unaccompanied foreign minors. In Cadiz, a provincial protocol exists to govern measures related to unaccompanied foreign minors and the determination of their age. This protocol acknowledges the significance of comprehensive database development, particularly in the initial days after admission to protection centers, as a substantial percentage (48%) of minors without identity documents are at risk of falling into vulnerable immigrant groups. These minors face potential exploitation, involvement in criminal activities, or sexual exploitation. By including them in the database of unaccompanied alien minors, effective identification becomes a valuable tool for implementing necessary protective measures and preventing fraudulent exploitation of their undocumented status, the use of fake identities, or the denial of their minor status.

#### 4.1.3. Children with Disabilities

Following the European Child Guarantee framework, this study examines the category of disabled children in Cadiz. The General Directorate of People with Disabilities, operating under the Regional Government of Andalusia, gathers annual data at the provincial level on children with a disability degree of 33% or higher. Notably, Cadiz is the third province in Andalusia with the highest number of children facing disabilities in 2022. This data sheds light on the specific challenges and needs of children with disabilities, facilitating a comprehensive understanding of child poverty within this category.

### 4.2. Essential Dimensions for Monitoring Child Poverty

#### 4.2.1. Children in Precarious Family Situations

One of the local monitoring projects that aligns with the objectives of this paper is conducted by the Municipal Delegation for Social Affairs of Cadiz City Council. The project involves a questionnaire-home interview conducted by social workers with families at risk of exclusion to assess their objective reality. The questionnaire covers housing, economic situation, employment, education and training, health, and social integration indicators. The survey results categorize families into levels of vulnerability ranging from low to extreme social exclusion based on a specific score for each level. This scale helps determine the appropriate measures and intensity of intervention [45]. During an interview for this paper, the coordinator of social programs and services for the elderly at the Municipal Delegation for Social Affairs shared that the project was launched in 2015 to improve the understanding of the challenges faced by families who require social services. The aim was to gain a more objective insight into their situation. Over time, the questions and indicators in the family surveys have been refined. The answers provide insights into the level of child poverty at the municipal level. The coordinator emphasized the importance of measuring the monetary benefits families receive and the daily expenses they incur. For example, two families may receive the same subsidy, but one may have higher expenses due to healthcare costs for an elderly dependent.

According to interviewees from the Márgenes y Vínculos Foundation, the foster care system provides data on the number of children experiencing severe deprivation and placed with foster families. They highlighted that there are no children experiencing homelessness under eight years old in Cadiz, as all such children are placed with foster families. Professionals use a comprehensive assessment system to identify deficits and skills in children, enabling better evaluation of their needs and foster care options. This measurement system is included in the “Intervention Protocol for the Management of the Foster Care Measure” of 2022, implemented by the Junta de Andalusia based on the recent Law on Childhood and Adolescence in Andalusia of 2021.

The Prosecutor’s Office for Children, which also deals with cases of child neglect, reported in the conducted interview that the most common situations related to child poverty that they handle include child abuse, interfamilial abuse, parental drug addiction, and poor housing conditions.

One of the youth organizations interviewed mentioned their collaboration with Cadiz City Council in 2022 through the CaixaProinfancia Program, aimed at assisting 50 vulnerable families affected by poverty and social exclusion. Indicators are used locally in Cadiz to identify families with the greatest need. These indicators encompass economic factors, education and schooling of children, health, housing, intra-family relations, and socio-cultural capital. Poverty rates among potential beneficiary families are determined based on the Spanish Multiple Effects Public Income Indicator (IPREM), which serves as a reference for providing aid, subsidies, and unemployment benefits in Spain. Lastly, the interviewed organization emphasized the importance of regular meetings among professionals to monitor the needs of children. Representatives from youth organizations, health centers, education centers, and the municipal social affairs service gather twice monthly for case management meetings. During these meetings, they discuss which families should be served under the program, considering the abovementioned criteria and indicators. Families consent for professionals to represent them, and health centers and schools share information on vaccinations and truancy.

#### 4.2.2. Violence

According to the interviewees, the implementation of National Law 8/2021 and Andalusian Regional Law 4/2021 represents a significant advancement in the protection of minors and the fight against child poverty. Law 8/2021 explicitly references the 2030 Agenda, with a particular focus on Goal 16.2, which aims to end abuse, exploitation, trafficking, violence, and torture of children. Interviewees responsible for minors in public administration institutions, such as schools, highlight that the law emphasizes the importance of informing them about potential risk situations that could harm a child’s personal, family, social, or educational development and well-being. Local protocols established by law are utilized to assess risk indicators. Spanish law 4/2021 introduces notable innovations, such as providing an information and indicator system derived from primary sources like the education and health systems. In collaboration with the Institute of Statistics and Cartography of Andalusia, these sources enable the measurement and understanding of the actual well-being of children and adolescents in Andalusia. This allows for identifying weaknesses, threats, strengths, and opportunities. The law’s motivation is to establish a common framework for interventions, inter-administrative actions, coordinated prevention actions, and their monitoring and evaluation.

Despite recognizing the need for indicators in Andalusian legislation on childhood and adolescence, UNICEF interviewees confirm that such indicators still need to be implemented. At the local level, interviewees widely use the Andalusian protocols SIMIA and VALORAME (See Appendix B). These tools, based on specific indicators, are deployed to identify signs of child abuse and measure its severity or intensity. Childcare professionals, such as teachers, school directors, and psychologists, complete the forms, which are then evaluated by municipal social services and juvenile court judges to determine the necessary support for the child. This tool aligns not only with the 2030 Agenda (SDG 16.2) but also with Council Recommendation (EU) 2021/1004, which establishes a European Child Guarantee and emphasizes that children in precarious family situations are exposed to various risk factors that may lead to poverty or social exclusion.

Local youth organizations working with children, addressing child abuse and campaigning for its prevention use indicator tables inspired by the protocols above. These tables consist of indicators related to monitoring the physical condition of children, behavioral or emotional indicators in the child, and behavioral and attitudinal indicators in the parents. Professionals conduct lectures and training sessions to raise awareness about child abuse and teach childcare workers how to recognize and respond to such cases.

#### 4.2.3. Nutrition and Food Security

In line with SDG Target 2.1 and the European Child Guarantee, access to healthy food and at least one nutritious meal per school day is considered an essential indicator for assessing the extent of child poverty. Following the implementation of this European recommendation, both at the national and regional levels, interviews conducted in schools revealed that this indicator is not only applied by local authorities based on specific criteria, such as the IPREM indicator mentioned earlier, or for families facing social exclusion or at risk of social exclusion. Additionally, an action plan has been implemented in schools to provide three meals—breakfast, lunch, and a snack—to ensure that children receive adequate nutrition. The provision of these meals in schools is aimed at addressing the nutritional needs of children and ensuring that they have access to healthy food throughout the school day. This approach aligns to combat child poverty by addressing one of the fundamental aspects of well-being and development yet it is noted that in most cases such initiatives treat the symptom yet not the root cause of the phenomenon. Moreover, implementing this action plan demonstrates a commitment to creating an inclusive and equitable education system that supports the overall well-being of children. Schools improve children’s health, concentration, and academic performance by providing nutritious meals. Additionally, this initiative helps alleviate the financial burden on families who struggle to provide regular and balanced meals for their children.

#### 4.2.4. Education

The importance of involving children and their families in improving their lives has been extensively discussed in the literature [48,50,51]. Children’s participation, which gives them a voice and the opportunity to evaluate goals and actions, is fundamental. As education is a critical element of measuring child poverty (SDG 4), a socio-cultural indicator (ISC) has been introduced at the local level in Cadiz, as reported by one of the schools interviewed in this work. This indicator provides a concrete assessment through repeaters, dropouts, truants, and students who graduate when appropriate. Additionally, it allows students, families, and teachers to evaluate their perception of the educational institution they are associated with through a satisfaction survey. Specific questions in the survey measure satisfaction with educational services, the climate and human interaction, and the educational projects and actions carried out by the institution.

School failure and dropping out are indicators of poverty and social exclusion. In Cadiz, a pilot project called ‘POPI’ has been implemented since 2022—the project aimed to identify families with truant children at risk of social exclusion. According to the City Council’s decree approving the project, funded by the EU Next Generation funds, a truancy protocol is initiated when a child has five unexcused absences. Strategies and actions are then developed with the families assigned to the treatment group from a holistic perspective, with the involvement of a family counselling team. The project is currently undergoing the revision of the administrative text on the prevention, monitoring, and control of truancy. This indicator of child poverty is monitored through multidisciplinary meetings of professionals, including educators, social workers, policymakers, and local police.

At the city level, Cadiz presented a local plan for 2018 that describes disadvantaged urban areas (ERACIS) and incorporates indicators to monitor child poverty. The plan includes disaggregated data by gender, age, and geography at the neighborhood level. Data for 2018 covers topics such as school absenteeism, illiterate and uneducated population, unemployment rate, underground economy rates, and the number of people with addiction problems, gender-based violence, and housing disrepair statistics. These indicators and monitoring efforts contribute to a comprehensive understanding of child poverty in Cadiz, allowing for targeted interventions and evaluating progress over time. By considering various dimensions of poverty, such as education, housing, and social integration, local authorities and professionals can develop strategies and allocate resources effectively to address the specific challenges children and families face.

#### 4.2.5. Social Networks

Child poverty can also be measured by examining access to internet platforms for communication between children, families, and schools. Indicators in this regard include having internet access at home, knowledge of how to use the platforms, and parental involvement in the process. At the regional level in Andalusia, two online educational platforms (e-learning) have been established to facilitate communication and learning between schools, children, and families. During interviews conducted in schools, including a compensatory school, the functioning of these platforms was explained, along with the internal efforts made to train families and children in their proper use. The absence of such platforms, particularly during the COVID-19 pandemic, would lead to a disconnect between families and schools, disproportionately affecting the poorest families.

Provincial-level studies in Andalusia investigate the relationship between socioeconomic status and the utilization of digital tools. These studies compare the use of educational platforms by families in compensatory schools with their use by families in standard schools. The findings suggest that families in the former category may not use these platforms for reasons such as needing to download them, more digital skills, or more time. Additionally, another study highlights how the limited education of these families contributes to their lack of digital communication.

The regional government of Andalusia also collects data on the percentage of families using digital applications and those who do not. From this information, it can be inferred that families in the latter group likely face signs of poverty, considering the indicators above.

#### 4.2.6. Community Engagement

Monitoring the existence and engagement of local children and youth councils or youth parliaments is crucial to assessing youth participation and empowerment. In Andalusia, this dimension is monitored annually at the provincial level by the prevention services of the territorial delegations of the Regional Ministry of Equality, Social Policy, and Mediation. These councils and parliaments serve as platforms for young people to voice their opinions, contribute to decision-making processes, and actively shape policies that affect their lives. By measuring the presence and number of such councils, Andalusia aims to evaluate how young people can engage, express their perspectives, and actively participate in matters that concern them.

Monitoring youth councils and parliaments aligns with the broader goal of promoting youth empowerment and democratic participation. These initiatives foster a sense of ownership, responsibility, and active citizenship among the youth population by providing young people with a platform to voice their ideas, concerns, and aspirations. The annual measurement of this dimension at the provincial level reflects a commitment to ensuring that young people have a voice in the decision-making processes that shape their communities. It acknowledges the importance of involving young people in policy discussions and recognizing their unique perspectives and experiences. Through monitoring youth councils and parliaments, Andalusia aims to create an inclusive and participatory environment where young people can contribute to developing policies that address their specific needs and aspirations. This approach not only empowers young people but also strengthens the overall democratic fabric of society by promoting active citizenship and intergenerational dialogue.

## 5. Conclusions

By examining the perspectives of interviewees in Cadiz, the paper sheds light on the various categories and dimensions of vulnerable children and the indicators used for local-level monitoring. The results emphasize the importance of prioritizing the most vulnerable children in line with the LNOB principle. The findings of this study reveal significant gaps in the monitoring of child poverty at the local level. While data exists at the national and regional levels, limited data is available at the provincial or municipal level, and what does exist needs to be updated and more accessible. Access to data sources, especially those collected by private statistical companies, is often restricted and comes at a cost, hindering comprehensive and up-to-date monitoring efforts. Furthermore, while there are projects and programs implemented at the local level to address child poverty, there needs to be more transparency in monitoring these initiatives. It remains unclear how children experiencing poverty are identified, how poverty is measured, and who is responsible for this monitoring. To improve local monitoring efforts, several recommendations emerge, namely:

- Incorporating direct social interaction and conversations with children and families to identify primary driving forces and prioritize relevant indicators in addressing the multidimensional aspects of child poverty.
- Efforts to translate the Sustainable Development Goals (SDGs) into regional legislation and local projects show promise. Recent legislation in Spain, specifically addressing child poverty monitoring, reflects a growing recognition of the importance of this issue.
- Local and regional authorities, mainly municipal social services, are crucial in identifying and addressing child poverty. Their proximity to the community allows for early detection of poverty situations and the provision of timely assistance to vulnerable children and their families.
- Collaboration among professionals working directly with children in poverty is essential for effective monitoring at the local level. Regular meetings and knowledge sharing between stakeholders can enhance the accuracy of indicators and measurements of child poverty.
- The active involvement of children and their families in the monitoring process is vital.

In regard to the latter, although the issue emerged from the interviews, stressing the need to monitor with (and not only about) involved children individuals, families and communities, it was not part of the current study. It is however noted as a key recommendation for future research and related practice in order to ensure sufficient inclusivity of monitoring exercises that are not merely focused to externals and intermediators, but by encompassing the actual children (and their communities) experiencing poverty. By soliciting the perspectives of the affected communities themselves on both emotional and material aspects of poverty, a more comprehensive understanding of their experiences can be obtained.

In conclusion, this research emphasizes the need for improved local monitoring of child poverty. By combining various methods and utilizing a comprehensive set of indicators, monitoring child poverty locally can offer valuable insights into the unique challenges children and families face in specific communities. This knowledge can inform targeted interventions, policy formulation, and resource allocation to effectively address child poverty and work towards building more equitable and inclusive societies. By addressing the identified gaps and implementing the recommendations, policymakers and stakeholders can enhance their understanding of child poverty, drive policy changes, and ultimately improve the well-being and outcomes of children and families impacted by poverty.

## Figures and Tables

**Table 1:** Indicators for monitoring child poverty, focusing on different dimensions and disaggregations.

Dimension of Child Poverty	Suggested Indicators
Income and material deprivation	household income; access to necessities; ownership of durable goods such as housing, transport, and electronics; percentage of households below the poverty line; average per capita income
Education	school attendance; dropout rates; educational attainment; access to educational resources (textbooks, computers, and internet)
Health and sanitation	infant mortality rates; malnutrition prevalence; access to healthcare services; access to sanitation facilities
Social inclusion	existence (in the area of living) of social networks; community engagement; participation in extracurricular activities; discrimination and harassment experienced by children based on gender, ethnicity, and other factors; access to social safety nets and support services; percentage of children living in single-parent households; homelessness rates among children; children staying in correctional facilities
Clothing	availability of warm clothing during cold weather; percentage of children without adequate clothing for all seasons; number of functional shoes and clothing items; access to clean and hygienic laundry facilities; access to affordable clothing; clothing donations received by charities
Housing	housing quality; overcrowding and housing density; access to clean and safe housing facilities; housing tenure and ownership status
Information	access to information technology (computer and/or internet); number of books available at home; access to newspapers, libraries, or other educational materials (online or printed); library resources available in the area of living; access to educational technology
Nutrition (food) and water	food security; malnutrition prevalence; level of stunting; access to clean and safe drinking water; availability of sufficient and nutritious food; number of meals missed due to financial constraints
Play	participation in extracurricular recreational activities; availability or access to toys, books, and recreational materials; access to safe play spaces
Other	child-headed households; incidence of child labor and exploitation; exposure to environmental hazards or other environmental externalities; access to transportation

**Table 2:** Key criteria for data collection of child poverty indicators.

Criteria for Data Collection	Definition
Core indicators	Collect data on the most relevant indicators. Indicators could be universal–equally applicable and pertinent across all countries or tailored to specific local challenges
Frequency	Collect data frequently enough. The correct frequency depends on the phenomenon being studies.
Promptness and availability	Ensure that data is cleansed and accessible in a timely manner; while also guaranteeing that metadata are publicly available.
Country coverage	Maximize comparability across countries, regions or cities when possible (noting that comparability does not entail a levelling of the unique, local circumstances)
Multi-dimensional and integrated	Merge data of the same individuals and households from different perspectives to monitor the multidimensional nature of deprivation (if allowed)
Cross-sectional and inter-temporal comparability	Collect data on the core indicators using a standardized approach that allows for comparisons within and across territories (e.g., countries, regions, municipalities), and over time. Core indicator definitions should be possibly harmonized with those of the SDGs.
Disaggregation	For example, factors like disability status, ethnicity, gender etc. should be considered, and ensure sample sizes are sufficient to permit disaggregation by these characteristics.
Population coverage	Aim at data at being fully representative–either through adequate sampling or complete population coverage.
Intra-household analysis	Fill data gaps within households and permit a richer understanding of intra-household dynamics.
Data quality	Maximize precision. Minimize sampling and non-sampling measurement error as well as data entry errors and data loss post collection.

**Figure A1:**
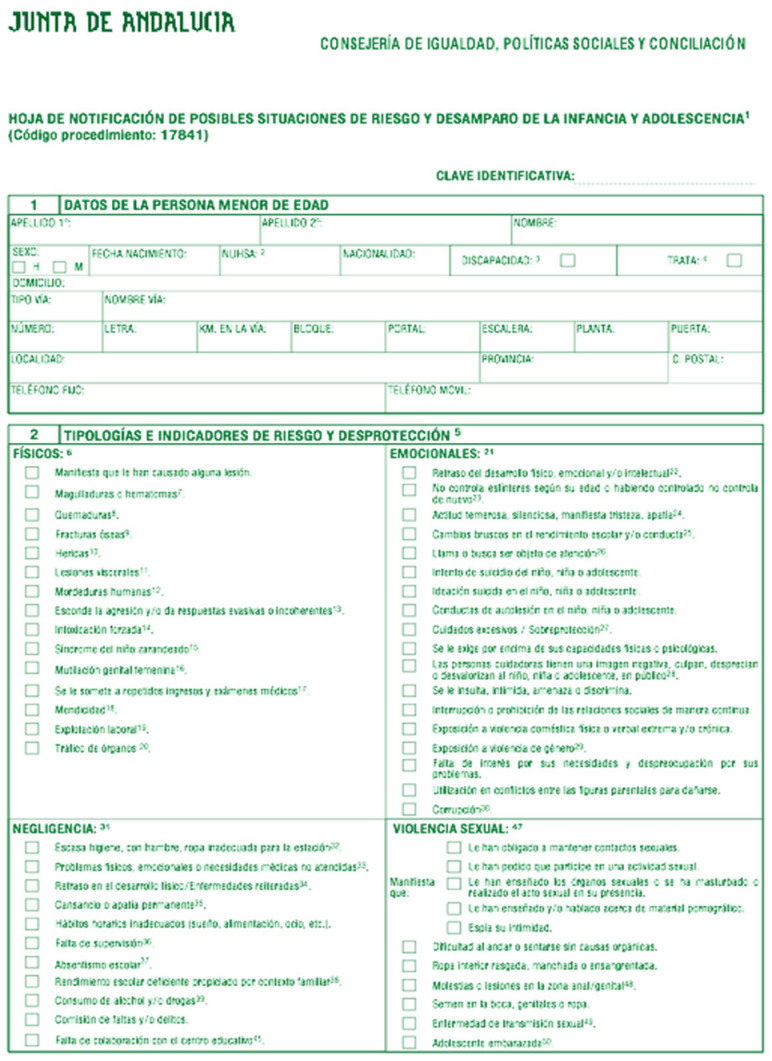
Protocols deployed to identify signs of child abuse and measure its severity or intensity.

## Data Availability

All data sources included in the analyses, tables and figures are referenced in the text.

## Appendix A

**Table A1:** List of stakeholders who were interviewed during the field visit in Cadiz (March 2023).

Role/Title	Organization
Director	Primary School Adolfo de Castro
Director	Secondary School Juan Luis Aragón
Director and Head of Studies	Secondary School Rafael Alberti
Prosecutor for Minors in Cadiz	Public Prosecutor Office
Coordinator, Social worker and Educator	Tierra de Todos Centre NGO
Coordinator-Educator and Director of foster care Program	Márgenes y Vínculos Fundación NGO
Coordinator of Social Affairs Programs of the Social Affairs Municipal Delegation, Delegate for Social Affairs of Cadiz City Council	Social Affairs Department of the Cadiz City Council
Social workers (2)	Social Work Unit of the Puerta del Mar Hospital
Head of the Child Protection Office	Territorial Delegation of Cadiz, Department of Social Inclusion, Youth, Families and Equality
Social workers (4)	Territorial Delegation of Cadiz, Department of Social Inclusion, Youth, Families and Equality

**Table A2:** List of stakeholders that were interviewed online (January–March 2023).

Role/Title	Organization
General Director of Child and Adolescent Rights	Ministry of Social Rights and 2030 Agenda, Spanish Government
Head of Childhood and Development Policies	UNICEF Spanish Committee
Responsible for Awareness and Childhood Policies in Andalusia Committee	UNICEF Spain
Sociologist	Social inclusion and knowledge management service, Observatory for Children and Adolescents of Andalusia
Professor of Economics	The National University of Distance Education, Spain
Professor at the Department of Sociology and Social Work	Comillas University, Madrid
Professor at the Faculty of Labour Relations	University of Cadiz
Technical advisor for children and education issues	Andalusian Ombudsman
Prosecutor for Minors in Cadiz	Public Prosecutor Office
Technical Coordination, Youth Department	Spanish Red Cross, Cadiz office
Social Inclusion Coordinator	The European Anti-Poverty Network, Andalusian Office
Postdoctoral Fellow	European Institute, London School of Economics

**Table A3:** List of stakeholders that were contacted but not interviewed.

Organization
IOM Spain (International Organization for Migration)
Save the Children Spain
Roma Community Foundation (Secretariado Gitano)
Members of Local Government of Cadiz
Local Police
Spanish Federation of Municipalities and Provinces (FEMP)
